# A multicenter open-label treatment protocol (HGT-GCB-058) of velaglucerase alfa enzyme replacement therapy in patients with Gaucher disease type 1: safety and tolerability

**DOI:** 10.1038/gim.2013.154

**Published:** 2013-11-21

**Authors:** Gregory M. Pastores, Barry Rosenbloom, Neal Weinreb, Ozlem Goker-Alpan, Gregory Grabowski, Gabriel M. Cohn, David Zahrieh

**Affiliations:** 1 Departments of Neurology and Pediatrics, New York University School of Medicine, New York, New York, USA; 2 Cedars-Sinai/Tower Hematology Oncology, Beverly Hills, California, USA; 3 University Research Foundation for Lysosomal Storage Diseases, Coral Springs, Florida, USA; 4 Lysosomal Research and Treatment Unit, Fairfax, Virginia, USA; 5 Children's Hospital Medical Center, Cincinnati, Ohio, USA; 6 Shire, Lexington, Massachusetts, USA

**Keywords:** antidrug antibodies, enzyme replacement therapy, imiglucerase, type 1 Gaucher disease, velaglucerase alfa

## Abstract

**Purpose:**

To evaluate the safety of velaglucerase alfa in patients with type 1 Gaucher disease who received velaglucerase alfa in the US treatment protocol HGT-GCB-058 (ClinicalTrials.gov identifier NCT00954460) during a global supply shortage of imiglucerase.

**Methods:**

This multicenter open-label treatment protocol enrolled patients who were either treatment naïve or had been receiving imiglucerase. Patients received intravenous velaglucerase alfa every other week at a dose of 60 U/kg (treatment naïve) or 15–60 U/kg (previously treated).

**Results:**

A total of 211 (including six treatment-naïve) patients were enrolled. Among the 205 previously treated patients, 35 (17.1%) experienced an adverse event considered related to study drug. Among the six treatment-naïve patients, one had an adverse event considered related to study drug. Infusion-related adverse events occurred in 28 (13.3%) of the 211 patients and usually occurred during the first three infusions. De novo, nonneutralizing, anti–velaglucerase alfa antibodies developed during treatment in one (<1.0%) previously treated patient and none of the treatment-naïve patients.

**Conclusion:**

The currently observed safety profile was consistent with those previously reported for imiglucerase and velaglucerase alfa phase III clinical trials. These results support the safety of initiating treatment with velaglucerase alfa or transitioning patients from imiglucerase therapy to velaglucerase alfa therapy.

## Introduction

Gaucher disease type 1 (GD1) is an autosomal recessively inherited lysosomal storage disease.^1^ Its pleotropic manifestations result from mutations in the gene encoding glucocerebrosidase (*GBA1*)^2^ and consequent dysfunction of the cognate enzyme. The resultant accumulation of glucosylceramide in the reticuloendothelial system leads to the major clinical features.^1^ Lipid-laden macrophages, called Gaucher cells, accumulate in various organs, including the liver, spleen, and bone marrow, resulting in cytopenias, hepatomegaly, splenomegaly, osteonecrosis, osteoporosis, and other bone lesions that occur by unclear molecular mechanisms.^1^

Worldwide, most patients with GD1 who are on treatment receive exogenous enzyme. Available enzyme therapies include imiglucerase (approved by the US Food and Drug Administration (FDA) in 1994 and by the European Medicines Agency in 1997), velaglucerase alfa (approved by the FDA and European Medicines Agency in 2010), and taliglucerase alfa (approved for adults by the FDA in 2012), all of which are generally administered intravenously every other week (EOW) at infusion centers, physician offices, or in a supervised home setting.^3,4,5,6^ Velaglucerase alfa enzyme therapy is now approved for the long-term treatment of adults and children with GD1 in >40 countries, including the United States, European Union member states, and Israel.^7^

Previous publications have reported the results of randomized, controlled clinical trials in patients with GD1 who received enzyme replacement therapy with imiglucerase^8,9^ or velaglucerase alfa.^7,10,11,12^ These trials were typically limited to patients who met specific eligibility criteria (e.g., regarding disease severity and involvement, previous treatment, presence of an intact spleen, and comorbidities) and enrolled between 15 and 95 patients. In contrast with these controlled clinical trials, the current report presents results from an observational study that involved a heterogeneous patient population with variable exposure to velaglucerase alfa. HGT-GCB-058 was an investigational new drug treatment protocol (ClinicalTrials.gov identifier NCT00954460) initiated at the request of the FDA in 2009. It enabled the provision of velaglucerase alfa, an investigational drug at the time, to patients with GD1 who were faced with an interruption, delay, or reduced dosage of imiglucerase. Patients who transitioned from imiglucerase were eligible to receive home therapy if they tolerated several velaglucerase alfa infusions in a physician-supervised setting. This study enabled us to evaluate the safety of velaglucerase alfa in a population of patients with GD1, which was larger and more heterogeneous than that typically included in clinical treatment trials for rare diseases.

## Materials and Methods

### Patients

The protocol population included US patients aged ≥2 years who had a documented (by enzyme and/or mutation analysis) diagnosis of GD1 and who were either treatment naïve (and requiring immediate initiation of treatment owing to the severity of their disease) or were currently receiving imiglucerase at prescribed or reduced doses or had stopped receiving imiglucerase. Eligible patients had no previous anaphylactic or anaphylactoid reaction to another enzyme therapy, including imiglucerase treatment, were not pregnant, and agreed to use a medically acceptable method of contraception throughout the study period. If new to treatment, eligible patients had at least one of the following GD-related abnormalities: anemia, splenomegaly (spleen palpable ≥2–3 cm below the left costal margin), thrombocytopenia, or palpable hepatomegaly.

### Protocol design and treatments

HGT-GCB-058 was a multicenter, open-label treatment protocol. Velaglucerase alfa, a human glucocerebrosidase, was derived from gene activation technology in the cell line HT-1080, a fibroblast-like derivative. Patients were enrolled from 9 September 2009 to 7 June 2010. Patients could withdraw informed consent to participate in the treatment protocol at any time and for any reason. Following FDA regulatory approval in February 2010, protocol patients were able to transition to commercial velaglucerase alfa. The final patient completed the protocol on 29 April 2011.

***Velaglucerase alfa treatment.*** The total every-4-weeks (Q4W) velaglucerase alfa dose was divided evenly and infused intravenously EOW at a maximum rate of 1 U/kg/min. Treatment-naïve patients received velaglucerase alfa infusions at a dose of 60 U/kg EOW. Patients transitioning from imiglucerase received velaglucerase alfa at doses between 15 and 60 U/kg EOW as follows: those previously receiving imiglucerase at 30–120 U/kg Q4W received velaglucerase at an equivalent dose; those who had experienced imiglucerase dose reductions because of supply constraints were eligible to receive velaglucerase alfa at a dose equivalent to their pre- or postreduction dose of imiglucerase, as determined by the investigator; and those who transitioned from a dose of imiglucerase <30 U/kg/month received an initial dose of velaglucerase alfa 15 U/kg EOW. Dose adjustments for clinical need were permitted for all enrolled patients based on physician discretion. Because the protocol design focused on velaglucerase alfa safety rather than on treatment efficacy, information on the preenrollment duration of imiglucerase interruption or dose reduction was not collected.

Patients transitioning from imiglucerase were eligible for home infusion following completion of the first three infusions of velaglucerase alfa at the clinical site with no infusion-related adverse events (AEs) and no treatment-related serious AEs (SAEs). Home infusions were administered by a qualified health professional under the direction of a physician or a home health agency. Quarterly evaluation visits to the clinic were required.

### Safety and tolerability

Safety data collected throughout the study period included physical examination, vital sign monitoring, clinical laboratory evaluation (hematology and clinical chemistry), assessment for anti–velaglucerase alfa antibodies, and monitoring for AEs. Vital signs were assessed at EOW dosing. Physical examinations, hematology, chemistry, and anti–velaglucerase alfa antibody assessments were done before the first dose and during quarterly site visits.

AEs were coded using the Medical Dictionary for Regulatory Activities (version 9.0) and rated by severity and potential relationship to study drug. Treatment-emergent AEs (TEAEs) were defined as those occurring on or after the time of the first infusion until 30 days after the last infusion. Infusion-related AEs were defined as those that began within 12 h after the start of an infusion and were judged to be possibly or probably related to study drug. Any deaths, SAEs, and reasons for early withdrawal also were recorded.

### Antidrug antibody assessment

Both anti-imiglucerase and anti–velaglucerase alfa antibodies were evaluated at screening, before the first dose of study drug. During treatment, anti–velaglucerase alfa antibodies were evaluated in serum samples collected at weeks 13, 25, 37, 51, and 65.

A panel of methods was used to evaluate the serum samples, using a tiered testing approach, for the presence of anti-imiglucerase or anti–velaglucerase alfa antibodies.^13^ A highly sensitive bridging electrochemiluminescence assay was used for sample screening, in which calibration curves were generated using a mouse monoclonal antibody that binds to imiglucerase and velaglucerase alfa with similar affinities. When a sample was screened positive, it was then tested using two confirmatory assays—a radioimmunoprecipitation assay to detect immunoglobulin (Ig) G antibodies and an indirect electrochemiluminescence immunoassay to detect IgE type antibodies. Confirmatory electrochemiluminescence assays for IgM and IgA type antibodies were also performed as needed. Samples confirmed positive for the presence of anti-imiglucerase or anti–velaglucerase alfa antibodies were further characterized using a neutralizing antibody assay. The neutralizing antibody assay is based on a colorimetric enzyme activity assay using a synthetic substrate 4-nitrophenyl-β-D-glucopyranoside and quantifies the ability of anti-imiglucerase or anti–velaglucerase alfa antibodies to inhibit their enzymatic activities in vitro.

### Statistical analyses

The analysis population included all enrolled patients who received at least a partial dose of the study drug. Results are presented within the treatment-naïve and previously treated cohorts. Analyses were descriptive in nature.

Prespecified subgroup analyses were performed to assess the safety profile stratified by the following baseline factors: sex; age (2–17 years (pediatric), ≥18 years (adult), and ≥65 years (geriatric)); anti-imiglucerase antibody status; splenectomy status; and disease severity as indicated by hemoglobin concentration and platelet count. Additionally, data from previously treated patients were analyzed within four velaglucerase alfa EOW dose groups: 15 U/kg (doses ≤22.5 U/kg), 30 U/kg (doses >22.5 U/kg and ≤37.5 U/kg), 45 U/kg (doses >37.5 U/kg and ≤52.5 U/kg), and 60 U/kg (doses >52.5 U/kg).

## Results

### Patient disposition and exposure to study drug

Of the 211 enrolled patients, six were treatment naïve and 205 had previously been treated with imiglucerase. A total of 189 (89.6%) patients completed the protocol, and 22 patients discontinued; all of these had been previously treated and the primary reasons for discontinuation were the following: withdrawal of consent (for any reason): 17 (8.1%); AE: 3 (1.4%; of these, 2 patients experienced mildly increased blood pressure considered possibly related to study drug and 1 patient had moderate infusion-related nausea; all resolved without any sequelae); termination from the protocol by the investigator: 1 (0.5%; patient was not compliant with infusion visits); and others: 1 (0.5%; patient moved out of the country and was therefore no longer eligible to participate).

The dose of velaglucerase alfa ranged from 58 to 60 U/kg EOW in the six treatment-naïve patients and ranged from 15.3 to 64.7 (median: 38.9) U/kg EOW in the 205 previously treated patients. The median duration of exposure to study drug was 15.1 weeks among treatment-naïve patients and 26.0 weeks among previously treated patients. The total number of infusions a patient received ranged from 1 to 38, with a total duration of treatment that ranged from 0.1 to 74.1 weeks. Treatment duration varied because of how enrollment occurred in this investigational new drug treatment protocol (designed to provide access to velaglucerase alfa during a period of imiglucerase supply shortage). Patients enrolled into the study when imiglucerase was no longer available to them and when patients and investigators deemed enrollment appropriate; therefore, the timing of enrollment varied by site and patient. Furthermore, patients left the study when commercial drug (imiglucerase or velaglucerase alfa) became available to them, which varied with location and individual patient insurance coverage, further contributing to the variation in treatment duration. Among the 205 previously treated patients, 187 (91.2%) did not experience an infusion-related AE or treatment-related SAE during the first three infusions and were thus eligible for home infusion; 54 (26.3%) received at least one home infusion.

### Baseline demographic and clinical characteristics

Baseline demographic and clinical characteristics are shown in **Table 1**. The median (range) age in the total population was 54.0 (6–89) years; 110 (52.1%) patients were women and 203 (96.2%) patients were white. Overall, 72 (34.1%) patients had undergone a previous splenectomy.

***Presence of antibodies at baseline.*** At baseline, none of the six treatment-naïve patients tested positive for anti-imiglucerase or anti–velaglucerase alfa antibodies (**Table 1**). Among the 205 patients previously treated with imiglucerase, 37 (18.0%) had detectible antibodies to imiglucerase at baseline, before exposure to study drug. The 37 patients with imiglucerase antibody positivity at baseline fell into two groups. Twenty-five patients did not have antibodies to velaglucerase alfa. Of these, two patients had anti-imiglucerase neutralizing antibodies (22% and 29% inhibition; **Supplementary Table S1** online). Twelve of 37 patients were antibody positive for both anti-imiglucerase and anti–velaglucerase alfa antibodies despite no previous exposure to velaglucerase; of these, nine patients had cross-reactive neutralizing antibodies. Percentage inhibition was essentially identical for the anti-imiglucerase and anti–velaglucerase alfa neutralizing antibodies (mean (S.D.), 63.3 (13.5) and 61.9 (12.2), respectively) and was attributable to antibody cross-reactivity. One patient had neutralizing antibodies to velaglucerase alfa but not to imiglucerase (24% inhibition).

### Safety and tolerability

The incidence of TEAEs varied among dose groups. Among the six treatment-naïve patients, three (50%) experienced at least one TEAE; of these, one patient experienced a TEAE considered related to study drug (an infusion-related episode of back pain). Among the 205 previously treated patients, 89 (43.4%) experienced at least one TEAE; 35 of 205 (17.1%) patients experienced an AE considered related to study drug and 27 of 205 (13.2%) patients experienced infusion-related AEs (**Table 2**). The TEAEs in most of these patients (treatment naïve, 3/3 (100%); previously treated, 78/89 (87.6%)) were mild or moderate in severity. The most frequently reported TEAEs were headache, nasopharyngitis, nausea, and fatigue (**Table 2**).

**Figure 1:**
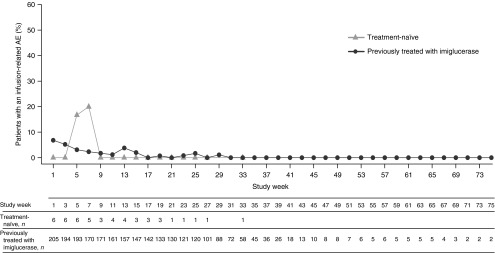
**Percentage of safety-assessment patient population experiencing infusion-related adverse events (AEs) by study week.** An infusion-related AE was defined as an AE that began either during or within 12 h after the start of the infusion and was judged as possibly/probably related to study drug.

Infusion-related AEs were reported in 28 (13.3%) of 211 patients and led to study discontinuation in three patients. No AEs of decreased blood pressure, hypotension, hypersensitivity, or anaphylactoid events were reported and no unexpected AEs occurred. Most infusion-related AEs occurred within the first 3 months of treatment (**Figure 1**) and nearly two-thirds of patients with infusion-related AEs experienced them during the first three infusions **(Supplementary Table S2** online). The proportions of patients who reported any TEAEs within a given 3-month period decreased as treatment progressed **(Supplementary Table S2** online). Of the 27 previously treated patients who experienced infusion-related AEs, 18 had infusion-related AEs during the first three infusions and 22 had infusion-related AEs during the first 3 months **(Supplementary Table S2** online).

SAEs were uncommon (**Table 2**), and only occurred in previously treated patients. A mild migraine SAE in the 60 U/kg dose group was considered possibly related to study drug. The other SAEs were classified as severe and unrelated to study drug. These included one case each of hip fracture, coronary artery disease and hypercholesterolemia, angina pectoris, cerebrovascular accident, bone pain and depression, and diverticulitis and bacterial peritonitis.

Discontinuations owing to AEs considered possibly or probably related to velaglucerase alfa occurred in 3 of 205 (1.5%) previously treated patients (mild blood pressure increase possibly related in two patients and moderate nausea probably related in one patient). No treatment-naïve patients discontinued owing to an AE.

### Clinical laboratory evaluations

Hemoglobin concentrations increased from a median of 9.25 g/dl (range: 8.2–13.3 g/dl; 92.5 (82–133) g/l) at baseline in treatment-naïve patients (mean increase: 1.68 g/dl (16.8 g/l) at week 13; *n* = 4) and were stable on average in previously treated patients. Platelet counts increased from a median of 94.0 (range: 57–232) × 10^9^/l at baseline in treatment-naïve patients (mean increase: 62.3 × 10^9^/l at week 13; *n* = 3) and were stable on average in previously treated patients. Values for other hematology parameters and serum chemistry analyses were unremarkable.

### Antibody development

During treatment, serum samples were evaluated for the presence of anti–velaglucerase alfa antibodies. Samples evaluated included those from 4 of 6 treatment-naïve patients and 163 of 205 previously treated patients. None of the treatment-naïve patients tested positive for anti–velaglucerase alfa antibodies at baseline, nor did they test positive for anti–velaglucerase alfa antibodies during treatment.

Among the 163 patients who had been previously treated with imiglucerase, received treatment for at least 13 weeks, and were assessable for development of anti–velaglucerase alfa antibodies during treatment, 31 patients were positive for anti-imiglucerase antibodies at baseline, including 10 who also were positive for anti–velaglucerase alfa antibodies **(Supplementary Table S3** online). Furthermore, nine patients who were positive for both anti-imiglucerase and anti–velaglucerase alfa antibodies at baseline remained positive for anti–velaglucerase alfa antibodies during the study, one patient (velaglucerase 60 U/kg dose group) who was positive for both anti-imiglucerase and anti–velaglucerase alfa antibodies at baseline became negative for anti–velaglucerase alfa antibodies during the study, and one patient (30 U/kg dose group) who was positive for anti-imiglucerase antibodies but negative for anti–velaglucerase alfa antibodies at baseline became positive for anti–velaglucerase alfa antibodies during the study. There was no apparent relationship between antidrug antibody status and the occurrence of TEAEs or infusion-related AEs (**Table 3**).

In summary, among the 167 patients with antibody assessments conducted after baseline (including four treatment-naïve and 163 previously treated patients), one patient (0.6%; velaglucerase 30 U/kg dose group), who had been previously treated with imiglucerase and was positive for anti-imiglucerase antibodies at baseline, developed anti–velaglucerase alfa IgG antibodies (but not IgE or neutralizing antibodies) during treatment.

### Subgroup analyses of TEAEs

Subgroup analysis by age (pediatric, adult, and geriatric groups) showed no substantive differences by age; however, the pediatric sample size was small (2/6 treatment-naïve and 6/205 previously treated patients were aged <18 years).

Subgroup analysis by previous splenectomy also showed no substantive differences between previously treated splenectomized and nonsplenectomized patients in overall AEs (45.1% and 42.5%, respectively), infusion-related AEs (9.9% and 14.9%, respectively), musculoskeletal and connective tissue AEs (16.9% and 9.0%, respectively), and general disorders and administration site conditions AEs (16.9% and 8.2%, respectively). Of the 10 patients who were positive for anti–velaglucerase alfa antibodies during the study, one had undergone splenectomy before the study. Differences observed in subgroup analyses by sex, baseline hemoglobin concentration, baseline platelet count, and positive anti-imiglucerase antibody status at baseline were unremarkable.

## Discussion

This investigational new drug treatment protocol enabled the provision of velaglucerase alfa to patients with GD1 who would otherwise have experienced a disruption or delay in receiving enzyme replacement therapy due to a global supply shortage of imiglucerase. Velaglucerase alfa was generally well tolerated in this large, clinically heterogeneous group of patients with GD1. The safety profile observed in this protocol was consistent with the profiles previously reported for imiglucerase,^14^ observed in the clinical development program for velaglucerase alfa,^7,10,12^ and reported in an early access velaglucerase alfa program from Israel.^15^ As described in the prescribing information, ~13.8% of patients receiving imiglucerase experienced related AEs.^16^ The most common AEs reported in patients receiving velaglucerase alfa were infusion-related reactions (any event considered related to and occurring within 24 h of infusion), seen in 51.9% of treatment-naïve patients and in 22.5% of patients previously treated with imiglucerase.

In the current study, most TEAEs were mild or moderate in severity and >90% of previously treated patients met the safety criteria for transition to home therapy after three infusions at the study site (clinic). Home therapy was at the discretion of clinicians and patients, and only 54 of 187 eligible patients actually received home therapy. Although the reasons for this were not available in the database, we believe this reflects a variety of factors, including lack of investigator experience with home therapy, lack of patient demand for home therapy when clinic access was convenient, and the low comfort level of attending physicians and nurses with home infusion of an investigational drug. In addition, a large proportion of patients in this study were aged ≥65 years and investigators may have been reluctant to start these patients on home therapy because these patients would not have been able to continue on home therapy under current Medicare rules once they transitioned to commercial drug.

Treatment with velaglucerase alfa in the current study was associated with a very low frequency of de novo development of anti–velaglucerase alfa antibodies, in agreement with results from phase III clinical trials, wherein approximately 1.2% of patients (both treatment naïve and those previously treated with imiglucerase) developed anti–velaglucerase alfa antibodies during treatment.^7,10,12^ The presence of a positive result for anti–velaglucerase alfa antibodies at baseline in 12 patients who had transitioned from imiglucerase and had no previous exposure to velaglucerase alfa suggests that there was cross-reactivity with anti-imiglucerase antibodies. The presence of baseline anti-imiglucerase antibodies is consistent with previously reported experiences with imiglucerase and alglucerase.^14,17^ Results from the current study suggest that anti-imiglucerase antibodies (and neutralizing antibodies in particular) have a high likelihood of cross-reacting with anti–velaglucerase alfa antibodies. Indeed, all patients who were positive for anti–velaglucerase alfa antibodies (12 at baseline and one who developed antibodies during treatment) were also positive for anti-imiglucerase antibodies at baseline and 10 of these had anti-imiglucerase neutralizing antibodies. Furthermore, of the 10 patients with anti–velaglucerase alfa neutralizing antibodies at baseline, all 10 were positive for anti-imiglucerase antibodies and 9 had anti-imiglucerase neutralizing antibodies at baseline. These findings indicate the presence of neutralizing antibodies against imiglucerase in several patients before velaglucerase treatment. These antibodies were cross-reactive with both imiglucerase and velaglucerase.

Despite the absence of specific efficacy objectives in the protocol design, the quarterly safety assessments provided results regarding the effects of velaglucerase alfa treatment on hemoglobin concentration and platelet count. The ability to assess the effects of velaglucerase alfa in treatment-naïve patients was limited by the small number of patients; however, the results suggested favorable effects, with improvement in both parameters in the treatment-naïve patient population **(Supplementary Tables S4** and **S5** online). Among previously treated patients, mean hemoglobin concentration and mean platelet counts were generally maintained, indicating that switching from imiglucerase to velaglucerase alfa at the same dose and regimen is associated with maintenance of efficacy for these parameters **(Supplementary Tables S4** and **S5** online). This is consistent with the results from a previous clinical trial in patients who transitioned from imiglucerase to velaglucerase alfa.^12^ Subgroup analyses according to baseline hemoglobin and platelet values did not show notably different results.

This study had several limitations. HGT-GCB-058 was a treatment protocol designed to provide velaglucerase alfa enzyme therapy to patients with GD1 during a period of imiglucerase shortage; it was not designed as a randomized, controlled clinical trial nor was it designed as an efficacy study to evaluate ongoing response to velaglucerase alfa treatment. Consequently, this study had minimal inclusion and exclusion criteria, resulting in a heterogeneous patient population that was weighted toward patients who had been previously treated with imiglucerase and a very small sample of treatment-naïve patients; subgroup analyses also had small sample sizes. Additionally, there was substantial variation in duration of treatment owing to the individual patient variations in timing of study entry and the timing of availability and transition to commercial therapy. However, the broad enrollment criteria allowed for a safety and tolerability assessment in a real-world population receiving typical enzyme replacement therapy.

Additional limitations of this study were the lack of information regarding the occurrence of AEs during previous therapy with imiglucerase (which were not collected and, indeed, not necessarily documented in primary source documents owing to the long-standing status of imiglucerase as an approved commercial drug) and the lack of information regarding how long the group of previously treated patients were off imiglucerase or on reduced doses of imiglucerase before initiation of velaglucerase alfa therapy. Furthermore, this study had no formal evaluation of either disease trajectory before study enrollment or efficacy outcomes during the trial and had a limited duration of safety observations (most patients received ≤9 months of treatment in this protocol) and a variable duration of drug exposure because patients transitioned onto commercially available velaglucerase alfa as it became available to them.

The current data support the safety of initiating treatment with velaglucerase alfa 60 U/kg EOW in patients with GD1 who are naïve to enzyme replacement therapy, in addition to showing the safety of transitioning patients from imiglucerase to velaglucerase alfa at the same dose as their previous imiglucerase dose. The safety profile of velaglucerase alfa observed across a broad range of patient ages is in agreement with that previously observed in controlled trials.^7,10,12^ In particular, velaglucerase was generally well tolerated in 52 patients who were aged ≥65 years, to date the largest reportable experience in a geriatric population receiving velaglucerase alfa. Depending on the patient's response and the recommendation of the treating physician, the observations from the treatment protocol further supports home therapy as a potential treatment option covered by medical insurance.

## Disclosure

O.G.-A. has received research support (Actelion, Shire, Genzyme/Sanofi, Amicus, Pfizer-Protalix Biotherapeutics), in addition to payments for consultancy (Actelion, Shire, Pfizer-Protalix Biotherapeutics) and speaker bureaus (Actelion, Genzyme/Sanofi, Shire). G.G. has received research support (Genzyme/Sanofi, Shire) and consultancy payments (Amicus, Genzyme/Sanofi, Pfizer, Shire, Synageva). G.M.P. has received research support (Actelion, Amicus, Biomarin, Genzyme/Sanofi, Protalix/Pfizer, Shire). B.R. has received research support (Genzyme/Sanofi) and has served on speakers bureaus (Genzyme/Sanofi, Shire). N.W. has received research support (Genzyme/Sanofi, Shire) and honoraria (Genzyme/Sanofi, Shire) and has served on advisory boards (Genzyme/Sanofi, Shire, Pfizer-Protalix Biotherapeutics) and speakers bureaus (Genzyme/Sanofi, Actelion, Pfizer). G.M.C. and D.Z. are employees of, and hold stock options in, Shire.

## Figures and Tables

**Table 1:**
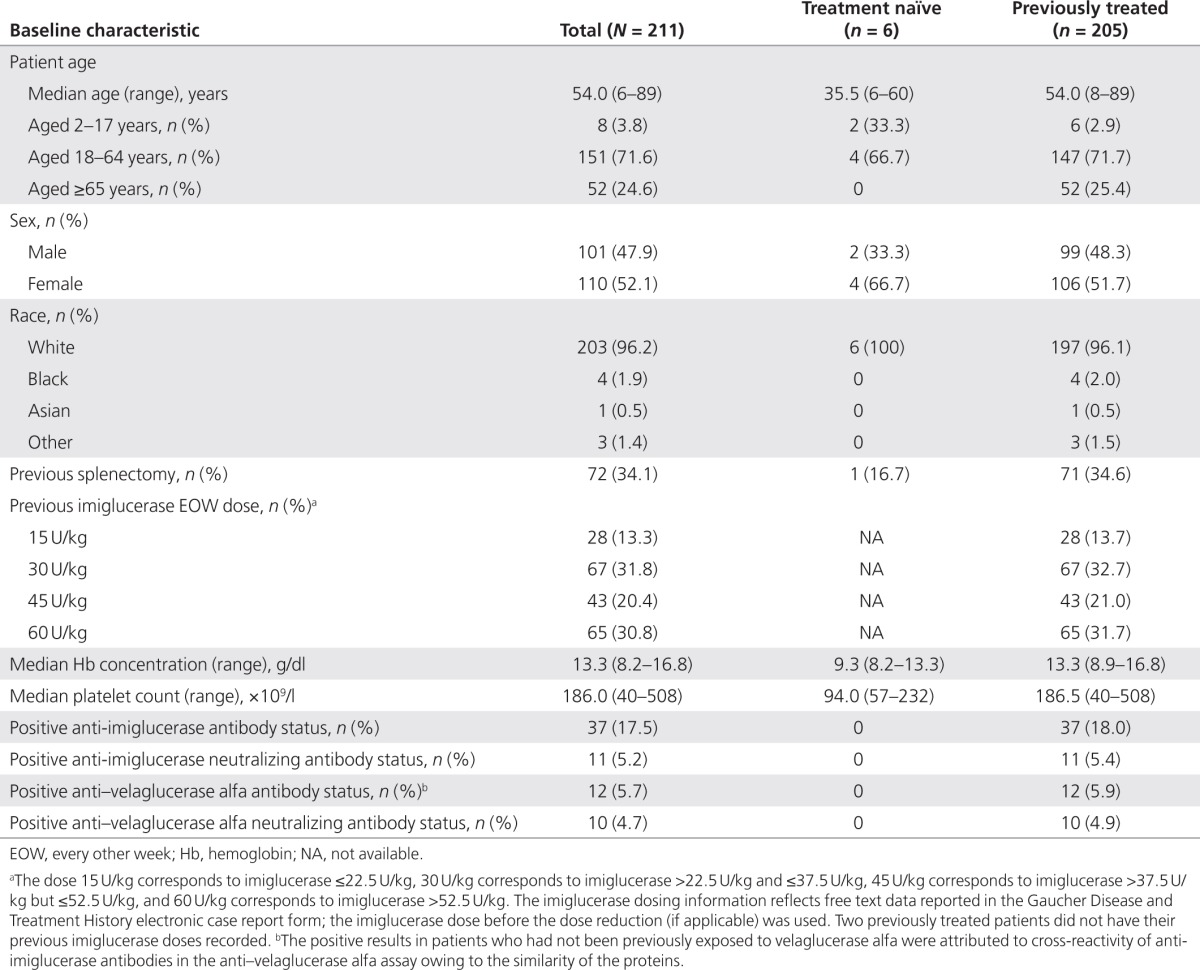
Baseline demographic and clinical characteristics

**Table 2:**
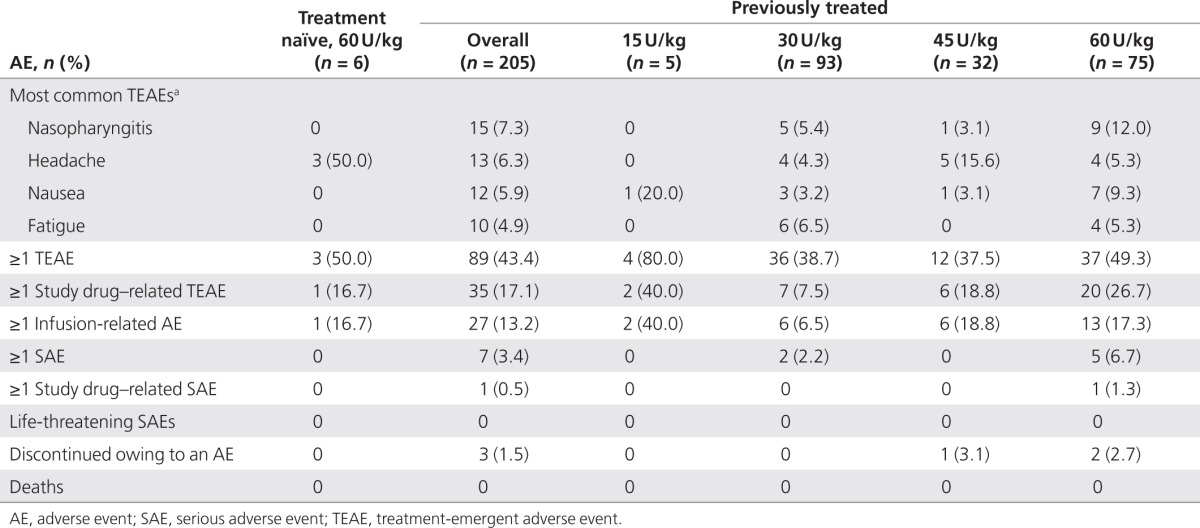
Summary of treatment-emergent AEs in safety-assessment patient population

**Table 3:**
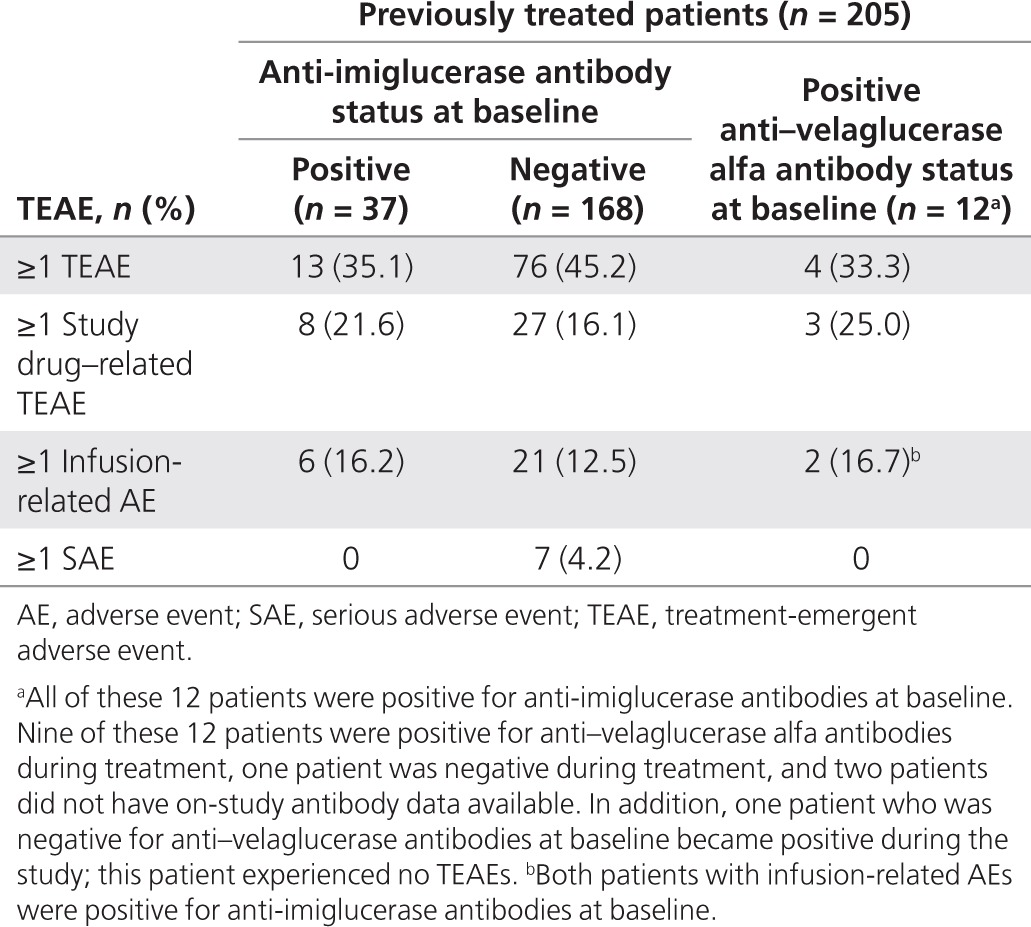
Summary of treatment-emergent AEs in previously treated patients by antidrug antibody status
