# Supplementary material for: A multicenter open-label treatment protocol (HGT-GCB-058) of velaglucerase alfa enzyme replacement therapy in patients with Gaucher disease type 1: safety and tolerability
Source: Genet Med. 2013 Nov 21;16(5):359–66. doi: 10.1038/gim.2013.154 (PMC4018500; doi:10.1038/gim.2013.154)
Supplement: Supplementary Table S1 [file gim2013154x1.doc]

**Supplemental Table S1.** Baseline neutralizing antibody results for individual patients

| **Patient identification** | **Anti-imiglucerase neutralizing antibodies, % inhibition** | **Anti-velaglucerase alfa neutralizing antibodies, % inhibition** |
| --- | --- | --- |
| 01*^a^* | 60 | 61 |
| 02 | 29 | NA |
| 03*^a^* | 80 | 76 |
| 04*^b^* | 60 | 53 |
| 05*^a^* | 52 | 49 |
| 06*^c^* | 74 | 71 |
| 07 | 22 | NA |
| 08*^a^* | 56 | 57 |
| 09*^a^* | NA | 24 |
| 10*^a^* | 44 | 53 |
| 11*^d^* | 88 | 86 |
| 12*^d^* | 52 | 51 |

NA, not applicable (patient did not have neutralizing antibodies).

^a^This patient did not experience any treatment-emergent adverse events. *^b^*This patient experienced one mild infusion-related reaction (sensation of foreign body). *^c^*This patient experienced five mild infusion-related reactions (flushing, hot flush, nausea, pruritus, urticaria). *^d^*This patient did not experience any infusion-related reactions.
