# Supplementary material for: A multicenter open-label treatment protocol (HGT-GCB-058) of velaglucerase alfa enzyme replacement therapy in patients with Gaucher disease type 1: safety and tolerability
Source: Genet Med. 2013 Nov 21;16(5):359–66. doi: 10.1038/gim.2013.154 (PMC4018500; doi:10.1038/gim.2013.154)
Supplement: Supplementary Table S2 [file gim2013154x2.doc]

**Supplemental Table S2.** Distribution of safety population patients with treatment-emergent adverse events during 3-month treatment windows

| **Patients** | **Treatment window** | | | | | |
| --- | --- | --- | --- | --- | --- | --- |
|  | **First 3 infusions** | **0–3 months** | **3–6 months** | **6–9 months** | **9–12 months** | **>12 months** |
| Treatment-naïve | | |  |  |  |  |
| *n* | 6 | 6 | 3 | 1 | 0 | 0 |
| Any TEAE, *n* (%) | 2 (33.3) | 3 (50.0) | 2 (66.7) | 0 | 0 | 0 |
| Infusion-related AE, *n* (%) | 1 (16.7) | 1 (16.7) | 0 | 0 | 0 | 0 |
| Previously treated | |  |  |  |  |  |
| *n* | 205 | 205 | 156 | 104 | 26 | 7 |
| Any TEAE, *n* (%) | 51 (24.9) | 70 (34.1) | 47 (30.1) | 16 (15.4) | 2 (7.7) | 2 (28.6) |
| Infusion-related AE, *n* (%) | 18 (8.8) | 22 (10.7) | 6 (3.8) | 1 (1.0) | 0 | 0 |

AE, adverse event; TEAE, treatment-emergent adverse event.
