# Supplementary material for: A multicenter open-label treatment protocol (HGT-GCB-058) of velaglucerase alfa enzyme replacement therapy in patients with Gaucher disease type 1: safety and tolerability
Source: Genet Med. 2013 Nov 21;16(5):359–66. doi: 10.1038/gim.2013.154 (PMC4018500; doi:10.1038/gim.2013.154)
Supplement: Supplementary Table S3 [file gim2013154x3.doc]

**Supplemental Table S3.** Summary of antibody status among 163 previously treated assessable patients

| **Antibody status** | **On-study anti-velaglucerase alfa antibody negative**  **(n=151)** | **On-study anti-velaglucerase alfa antibody positive**  **(n=10)** | **On-study anti-velaglucerase alfa IgG antibody positive**  **(n=10)** | **On-study anti-velaglucerase alfa IgE antibody positive**  **(n=1)** | **On-study anti-velaglucerase alfa neutralizing antibody positive**  **(n=8)** | **On-study anti-velaglucerase alfa antibody missing**  **(n=2)** |
| --- | --- | --- | --- | --- | --- | --- |
| Baseline anti-imiglucerase negative and anti-velaglucerase alfa antibody negative (n=132) | 130 | 0 | 0 | 0 | 0 | 2 |
| Baseline anti-imiglucerase negative and anti-velaglucerase alfa antibody positive (n=0) | 0 | 0 | 0 | 0 | 0 | 0 |
| Baseline anti-imiglucerase positive and anti-velaglucerase alfa antibody negative (n=21) | 20 | 1 | 1 | 0 | 0 | 0 |
| Baseline anti-imiglucerase positive and anti-velaglucerase alfa antibody positive*^a^* (n=10) | 1 | 9 | 9 | 1 | 8 | 0 |

EOW, every other week; IG, immunoglobulin.

*^a^*The positive results in patients who had not been previously exposed to velaglucerase alfa were attributed to cross-reactivity of anti-imiglucerase antibodies in the anti-velaglucerase alfa assay owing to the similarity of the proteins.
