# Supplementary material for: A multicenter open-label treatment protocol (HGT-GCB-058) of velaglucerase alfa enzyme replacement therapy in patients with Gaucher disease type 1: safety and tolerability
Source: Genet Med. 2013 Nov 21;16(5):359–66. doi: 10.1038/gim.2013.154 (PMC4018500; doi:10.1038/gim.2013.154)
Supplement: Supplementary Table S4 [file gim2013154x4.doc]

**Supplemental Table S4.** Mean ± standard deviation change from baseline in hemoglobin concentration

| **Baseline Hb subgroup** | **Week 13** | **Week 25** | **Week 37** | **Week 51** | **Week 65** |
| --- | --- | --- | --- | --- | --- |
| Treatment-naïve |  |  |  |  |  |
| Males 9–11 g/dL | 2.20  *n* = 1 | 4.80  *n* = 1 | NA | NA | NA |
| Females <9 g/dL | 2.40 ± 0.283  *n* = 2 | NA | NA | NA | NA |
| Females ≥11 g/dL | −0.30  *n* = 1 | NA | NA | NA | NA |
| Previously treated |  |  |  |  |  |
| Males 9–11 g/dL | 1.30  *n* = 1 | 1.30  *n* = 1 | NA | NA | NA |
| Males 11–12 g/dL | −0.35 ± 0.495  *n* = 2 | −0.80  *n* = 1 | NA | NA | NA |
| Males ≥12 g/dL | 0.15 ± 0.860  *n* = 66 | −0.01 ± 1.008  *n* = 51 | −0.37 ± 1.400  *n* = 15 | 0.66 ± 0.826  *n* = 5 | 1.47 ± 0.666  *n* = 3 |
| Females 9–11 g/dL | 0.26 ± 0.635  *n* = 5 | 0.42 ± 0.988  *n* = 5 | NA | NA | NA |
| Females ≥11 g/dL | 0.09 ± 0.793  *n* = 76 | 0.00 ± 0.741  *n* = 55 | 0.07 ± 0.433  *n* = 12 | −0.15 ± 0.354  *n* = 2 | −0.50  *n* = 1 |

Hb, hemoglobin; NA, not applicable.
