# Supplementary material for: A multicenter open-label treatment protocol (HGT-GCB-058) of velaglucerase alfa enzyme replacement therapy in patients with Gaucher disease type 1: safety and tolerability
Source: Genet Med. 2013 Nov 21;16(5):359–66. doi: 10.1038/gim.2013.154 (PMC4018500; doi:10.1038/gim.2013.154)
Supplement: Supplementary Table S5 [file gim2013154x5.doc]

**Supplemental Table S5.** Mean ± standard deviation change from baseline in platelet counts

| **Baseline platelet count subgroup** | **Week 13** | **Week 25** | **Week 37** | **Week 51** | **Week 65** |
| --- | --- | --- | --- | --- | --- |
| Treatment-naïve |  |  |  |  |  |
| 60–100 × 10^9^/L | 58.0 ± 9.90  *n* = 2 | NA | NA | NA | NA |
| ≥120 × 10^9^/L | 71.0  *n* = 1 | NA | NA | NA | NA |
| Previously treated |  |  |  |  |  |
| <60 × 10^9^/L | 19.0  *n* = 1 | 8.0  *n* = 1 | NA | NA | NA |
| 60–100 × 10^9^/L | 0.6 ± 19.51  *n* = 5 | 14.4 ± 12.52  *n* = 5 | 18.0 ± 9.90  *n* = 2 | NA | NA |
| 100–120 × 10^9^/L | 25.1 ± 27.29  *n* = 14 | 26.1 ± 37.84  *n* = 13 | 50.8 ± 78.34  *n* = 4 | 17.0 ± 21.21  *n* = 2 | 4.0  *n* = 1 |
| ≥120 × 10^9^/L | 10.0 ± 39.19  *n* = 122 | 20.4 ± 52.96  *n* = 91 | 3.5 ± 40.29  *n* = 20 | 15.0 ± 23.72  *n* = 5 | 35.0 ± 23.52  *n* = 3 |

NA, not applicable.
